# United States radiological health activities: inspection results of mammography facilities

**DOI:** 10.2349/biij.3.2.e35

**Published:** 2007-04-01

**Authors:** DC Spelic, RV Kaczmarek, M Hilohi, S Belella

**Affiliations:** 1 Division of Mammography Quality and Radiation Programs, Food and Drug Administration, Rockville, Maryland, United States; 2 SFA, Inc., Crofton, Maryland, United States

## Abstract

**Purpose:**

The Mammography Quality Standards Act (MQSA) was enacted in 1992 to set national standards for high-quality mammography, including standards for mammographic X-ray equipment, patient dose, clinical image quality, and related technical parameters. The MQSA also requires minimum qualifications for radiologic technologists, interpreting physicians and medical physicists, mandates acceptable practices for quality-control, quality-assurance, and requires processes to audit medical outcomes. This paper presents the findings of MQSA inspections of facilities, which characterize significant factors affecting mammography quality in the United States.

**Materials and Methods:**

Trained inspectors collected data regarding X-ray technical factors, made exposure measurements for the determination of mean glandular dose (MGD), evaluated image quality, and inspected the quality of the film-processing environment. The average annual facility and total U.S. screening exam workloads were computed using workload data reported by facilities.

**Results:**

Mammography facilities have made technical improvements as evidenced by a narrower distribution of doses, higher phantom-film background optical densities associated with higher phantom image-quality scores, and better film processing. It is estimated that approximately 36 million screening mammography exams were conducted in 2006, a rate that is almost triple the exam volume estimated for 1997. Digital mammography (DM) is now in use at approximately 14% (1,191 of 8,834) of MQSA-certified mammography facilities. The results indicate that DM can offer lower dose to the patient while providing comparable or better image quality.

## INTRODUCTION

The Mammography Quality Standards Act (MQSA) of 1992 was enacted to set national standards for high-quality mammography and ensure that clinical facilities in the U.S. meet those standards. In 1995, the Food and Drug Administration (FDA) initiated a program of inspections of the then approximately 10,000 mammography facilities to assess compliance with the MQSA standards. Trained inspectors collected exposure and technique data to determine radiation dose, evaluated phantom image quality, and tested the film processing environment to ensure that clinical mammograms were developed appropriately. The facility’s medical records were also evaluated for compliance with quality control and quality assurance standards, appropriate medical-audit processes, and for proper documentation of the professional qualifications of interpreting physicians, radiologic technologists, and medical physicists. Facilities that do not meet these standards must respond with an acceptable corrective plan or face legal action.

The specific MQSA technical standards for X-ray equipment, patient dose, and image quality were motivated in part by studies between the 1970s and the early 1990s [1,2] that documented the broad range of technical performance by mammography facilities. For example, although mammography quality had improved substantially as a result of better equipment performance through dedicated screen and film combinations, improved film processing, and the use of grids, other technical parameters- dose, background film optical density, and image quality indicators- still showed a broad range of values. The American College of Radiology (ACR) had initiated an accreditation program for mammography, and facilities could also be accredited through their state or become certified with the Health Care Financing Administration (HCFA). However in 1992, 23% of mammography facilities carried no credentials from a recognised professional organisation [2]. A subsequent study by Suleiman [3] et al. in 1999 documented improvements after three years of MQSA inspections for most areas of technical performance. This report discusses further trends in the practice of mammography after 11 years of MQSA inspections, and it focuses on technical indicators of quality and trends in the rate at which the U.S. population is screened for breast cancer.

Prior to the early 2000s mammography in the U.S. was based essentially on screen-film (SF) technology. Digital mammography (DM) was first approved for clinical use in the U.S. in 2000 and is now offered in 14% of MQSA-certified facilities. It has unique technical advantages over conventional screen-film technology by separating the technology for capturing images from the media for viewing and storing them. A disadvantage, however, is that unlike film, whose inherently limited sensitometric range of exposure acts to constrain the dose to the patient, DM equipment is capable of producing images of acceptable quality for a broad range of doses [4]. Nevertheless, studies [5,6] indicate the potential for DM to offer a lower dose than SF technology, and the extensive ACR Imaging Network-Digital Mammographic Imaging Screening Trial (ACRIN-DMIST) study comparing DM and SF concluded that DM is clinically superior for patients under the age of 50 years, premenopausal or perimenopausal patients, and patients with radiographically dense breasts, but is otherwise comparable in overall diagnostic accuracy for screening for breast cancer [7]. This paper also compares inspection findings for DM with those of conventional SF imaging and discusses the impact of DM on general practice.

Finally, although some studies question the benefit of population-wide mammography screening [8], it is generally accepted that there is benefit to the patient over and above the radiation and other risks involved [9,10,11]. This paper also examines facility annual screening workloads and provides estimates for total annual exam volumes in the U.S.

## EQUIPMENT AND PROCEDURES

Each MQSA-certified inspector is required to pass a series of three training courses provided by the FDA and complete additional field testing prior to conducting MQSA inspections independently. Each inspector was provided all the necessary equipment to make radiation dosimetry measurements, evaluate image quality and film processing quality, and inspect the processing darkroom environment. Exposure measurements for the determination of beam quality and mean glandular dose (MGD) were made with the MDH model 1015 (Radcal Corporation, Monrovia, CA) survey meter equipped with the 10X5-6M mammography ionisation chamber. The radiation meter and ionisation chamber were calibrated annually by the FDA’s X-ray calibration facility, which is accredited by the National Voluntary Laboratory Accreditation Program (NVLAP). Exposure measurements were done with a standard mammography phantom having radiographic attenuation properties equivalent to that of a 4.2-cm compressed breast composed of 50% glandular and 50% adipose tissue. Beam quality (half-value layer) was determined for the clinically configured kVp using type 1145 aluminum. MGD in this standard breast model was then computed using conversion factors derived by Wu, Barnes and Tucker [12]. The phantom also contains three sets of image-quality test objects: fibril-like objects, speck groups that simulate micro-calcifications, and mass-like objects, and it is commercially available (model 156 mammography accreditation phantom, Gammex RMI, Inc., Middleton, WI). A radiograph of the phantom was acquired using the same technical factors as those used for dosimetry data collection, and it was then evaluated for appropriate background optical density and acceptable image quality. MQSA requirements for phantom film image quality include a minimum background optical density of 1.2 and minimum scores for the three groups of test objects (including artefact subtraction): four fibers out of a possible score of six, three speck groups out of a possible five groups, and three masses out of a possible score of five. If a phantom radiograph failed for one or more of the test objects, a second radiograph was scored to confirm the assessment prior to citing the facility as non-compliant. After May 2006, MQSA inspectors no longer measured mammographic phantom doses themselves but instead captured dose values documented in the reports of the required annual medical physics surveys. During the transition away from independent dose measurements by MQSA inspectors, the FDA conducted a comparison study and validated (p < 0.001) the equivalence of the two means of assessing the dose in the standard breast.

Film processing quality was evaluated using the Sensitometric Technique for the Evaluation of Processing (STEP) [3,13]. A reference automatic film processor was configured for processing a selected test film according to the specifications recommended by the film manufacturer. The same film type was then distributed to all MQSA inspectors along with sensitometers calibrated to an FDA reference sensitometer, and densitometers that were calibrated to the National Institute of Standards and Technology (NIST) reference standard densitometry step tablet (SRM 1001). A relative speed value was determined for the facility’s film processor based on a comparison of optical densities from the test film processed at the facility versus the same film when processed in the FDA reference film processor. A processing speed of 100 was assigned if the tested film processor was operating in close agreement with film manufacturer specifications, whereas speeds greater than 120 or less than 80 (for standard cycle processing) indicate substantial deviation from acceptable film-processing levels. A film processing speed below 80 indicates substantial under-processing of the film and a facility could be motivated to compensate by increasing exposure. For this reason, MQSA regulations require a minimum film processor speed of 80. Extended cycle processing, where the film is developed over a longer period of time (a total development time of approximately 3 minutes as opposed to 90 seconds typically for standard cycle), results in a STEP processing speed of approximately 130 or greater depending on the processor and the film brand and type. MQSA requires extended cycle film processors to have a minimum processing speed of 100. MQSA has not specified a maximum permissible film processor speed for either processing cycle.

An undeveloped radiograph of the phantom was used to evaluate the darkroom environment for sources of radiographic fog. The film was placed in an area of the darkroom where mammography film is routinely handled, and then it was bisected so that approximately half of the latent image of the phantom was exposed to ambient darkroom conditions for 2 minutes. The film was then developed and inspected for an increase in the background optical density. If a distinct area of higher optical density was observed on the exposed side of the border, then the inspector determined the net increase in optical density across the border. MQSA requires that facilities maintain darkroom fog levels of net optical density not greater than 0.05.

MQSA-certified clinical facilities began reporting annual screening mammography workloads in 1997. Facilities were asked to provide annual workload rates during their initial application for mammography accreditation and during subsequent certification renewal. Average facility and total U.S. screening workloads were then derived using figures for the total number of certified mammography facilities. It was assumed that the workload sample set reported to FDA for each year, ranging from 7% to nearly 30% of all certified facilities, is representative of the U.S. state of practice.

## RESULTS

**Figure 1:**
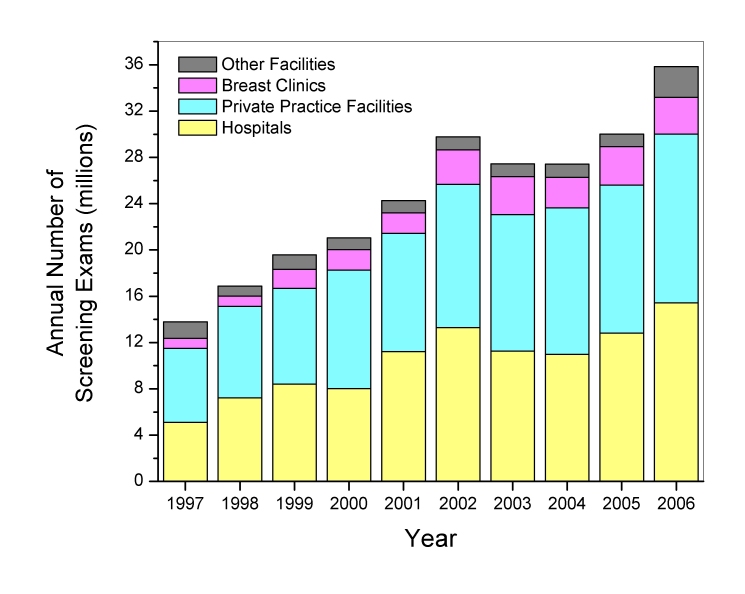
Total U.S. screening mammography annual examination workload, and contributions by facility type. Standard errors for the total annual estimates ranged between 3% and 12%.

**Figure 2:**
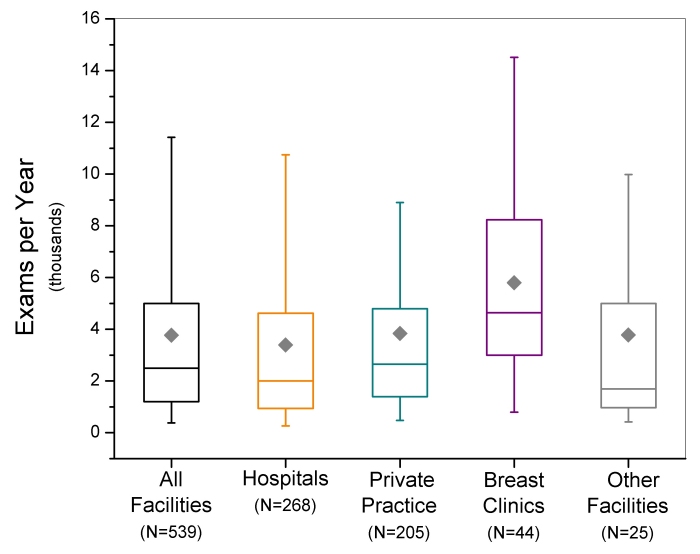
Box-whisker plots of facility screening mammography workloads reported between January 2006 and October 2006. Box bottom and top borders are 25^th^ and 75^th^ percentiles respectively, and box contents are 50^th^ percentile (line) and mean value (diamond). Whiskers indicate 5^th^ and 95^th^ percentiles.

**Figure 3:**
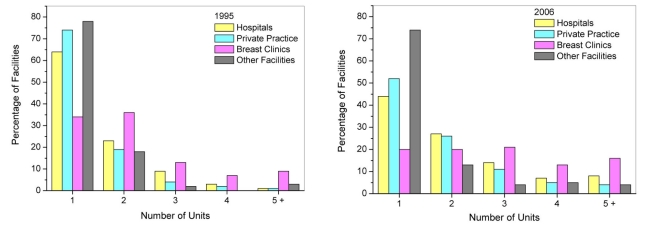
Distributions for 1995 (left) and 2006 (right) of the number of mammography units per facility used for mammography examinations by facility type.

Between 1997 and 2006, the total number of screening mammography exams performed annually in the U.S. increased linearly (r^2^=0.91) from approximately 13.8 ± 1.6 (mean ± standard error) million exams to 35.8 ± 1.9 million exams (Figure 1). Figure 1 also displays the contribution to the total U.S. screening workload for specific facility types: hospitals, private practice facilities such as outpatient radiology facilities, dedicated breast clinics, and facilities that were classified as ‘other’ if they did not meet the criteria for the preceding three facility categories. The calculations do not account for additional contributions from a small number (approximately 30) of the Department of Veterans Affairs facilities that may perform mammography but are not required to be compliant with MQSA regulations. Hospitals and private practices, which from 1995 to 2006 constituted approximately 42% and 48% of all certified mammography facilities respectively, consistently contributed the majority (over 80 percent) of screening exams. Dedicated breast clinics, which account for less than 6% of all mammography facilities, were found to consistently have the highest average facility screening exam workload of all identifiable facility types (excluding ‘other’) (Figure 2). All types of facilities increased their number of mammography units (Figure 3), which is consistent with the observation that while the number of certified mammography facilities has actually decreased since 1995 from approximately 10,000 facilities to just over 8,800 facilities, the total number of exams in the U.S. has increased substantially.

### Dose and Image Quality

**Figure 4:**
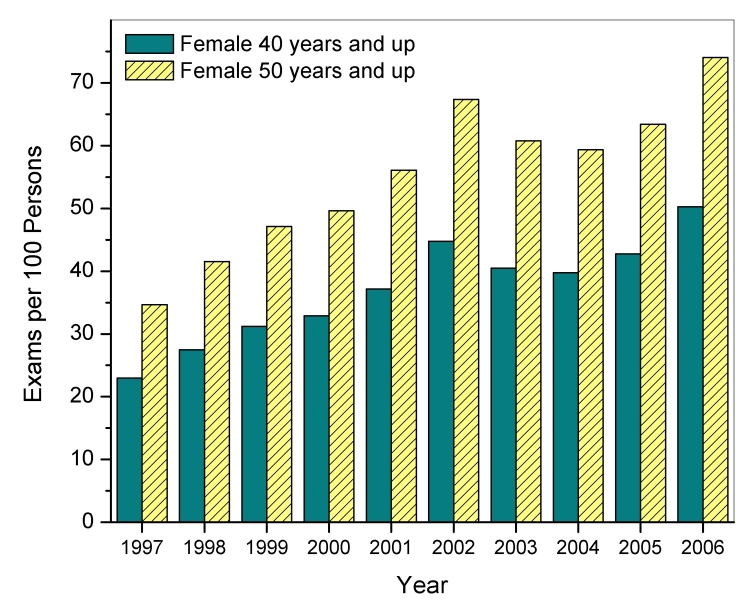
Box-whisker plots of mean glandular dose from MQSA inspections conducted in 1995 and 2006. Dose values for 1995 are those determined by the MQSA inspector. Values for 2006 are those reported in the facility medical physics survey report. Box bottom and top borders are 25^th^ and 75^th^ percentiles respectively, and box contents are 50^th^ percentile (line) and mean value (diamond). Whiskers indicate 5^th^ and 95^th^ percentiles.

**Figure 5:**
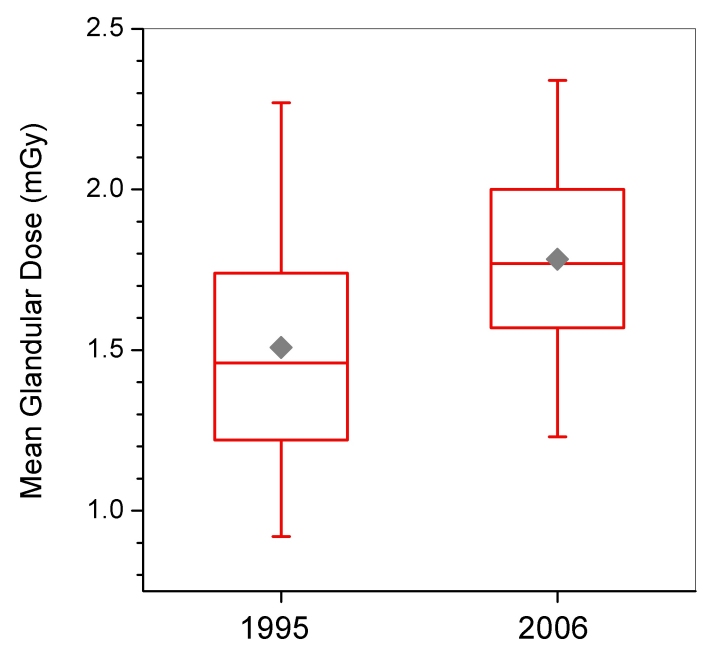
Distributions of mammography unit clinically selected tube potential (kVp) for 1995 and 2006 MQSA inspections.

Tables 1 and 2 summarize statistics for selected technical aspects of mammography in the U.S. for 1995 and 2006. During this time period there was a statistically significant increase in average MGD (p < 0.001) from 1.51 mGy to 1.78 mGy (Tables 1 and 2). The distribution of doses (Figure 4) about the mean decreased during the same time period, and this trend was observed across every type of facility. Dose is partly determined by the selected tube potential (kVp), and although the mean clinically selected tube voltage (kVp) did not change appreciably between 1995 and 2006, the distribution has narrowed (Figure 5). Over 90% (7,865 of 8,586) of inspected mammography units are now using either 25 or 26 kV, whereas in 1995 over 25% (3,068 of 11,697) of inspected units were operated at 27 kV or higher. The standard deviation for kVp decreased by almost half for every type of facility except breast clinics, which had the narrowest distribution in 1995. Half-value layer (HVL) followed a trend similar to that for kVp. The mean for HVL changed very little across all types of facility since 1995, but private practice sites had a standard deviation for HVL in 2006 that was less than half of the standard deviation for 1995.

**Table 1:** MQSA inspection results for selected technical parameters: January to December, 1995.

*Unless otherwise indicated, tabulated values are: N / **Mean** / SD*	**Hospitals**	**Private Practice**	**Breast Clinics**	**Other facilities**	**ALL**
Mean Glandular Dose^a^ (mGy)	5715/**1.50**/0.42	4959/**1.51**/0.42	730/**1.47**/0.39	293/**1.58**/0.44	11697/**1.51**/0.42
kVp	5732/**25.9**/1.2	4977/**26.0**/1.4	734/**25.9**/1.0	293/**25.9**/1.1	11736/**26.0**/1.2
HVL (mm Al)	5732/**0.32**/0.03	4977/**0.33**/0.05	734/**0.33**/0.02	293/**0.32**/0.02	11736/**0.33**/0.04
Phantom Image Background OD	5732/**1.43**/0.22	4977/**1.42**/0.23	734/**1.44**/0.21	293/**1.41**/0.23	11736/**1.42**/0.23
Fibers^b^	5729/**4.6**/0.6	4923/**4.5**/0.6	731/**4.6**/0.6	293/**4.5**/0.7	11676/**4.5**/0.6
Specks^b^	5729/**3.7**/0.5	4923/**3.7**/0.5	731/**3.7**/0.5	293/**3.7**/0.6	11676/**3.7**/0.5
Masses^b^	5729/**3.6**/0.6	4923/**3.6**/0.6	731/**3.7**/0.5	293/**3.6**/0.6	11676/**3.6**/0.6
Total Net Score^c^	5705/**11.5**/1.1	4955/**11.4**/1.1	732/**11.6**/1.0	290/**11.4**/1.1	11682/**11.5**/1.1
Film Processing: % < 80^d^	4.5 (80/1777)	3.4 (60/1742)	1.8 (2/109)	8.0 (7/88)	4.0 (149/3717)
Darkroom Fog: % > 0.05 OD^e^	10.5 (352/3365)	12.1 (381/3156)	8.8 (29/328)	8.8 (16/181)	11.1 (778/7030)
% Phantom Images with artifacts	62.2 (3565/5729)	57.1 (2812/4923)	56.9 (416/731)	63.5 (186/293)	59.8 (6979/11676)

a Dose was calculated from data acquired by the MQSA inspector.

b Object score is reported without artifact subtraction.

c Score includes artifact subtraction.

d Percentage of standard-cycle film processors with a speed less than 80, as determined by the STEP method.

e Percentage of facilities found to have darkroom fog greater than 0.05 net optical density.

**Table 2:** MQSA inspection results for selected technical parameters: January to September 2006

*Unless otherwise indicated, tabulated values are:N**/Mean**/ SD*	**Hospitals**	**Private Practice**	**Breast Clinics**	**Other facilities**	**ALL**	**Digital units^a^**	**Screen-FilmUnits**
Mean Glandular Dose^b^ (mGy)	4776/**1.78**/0.33	3681/**1.79**/0.35	806/**1.81**/0.33	359/**1.82**/0.31	9776/**1.78**/0.34	890/**1.63**/0.39	8542/**1.80**/0.33
kVp	4271/**25.5**/0.7	3328/**25.5**/0.7	653/**25.5**/0.7	334/**25.5**/0.7	8586/**25.5**/0.7	N/A	8586/**25.5**/0.7
HVL (mm Al)^c^	2227/**0.33**/0.02	1814/**0.33/**0.02	418/**0.32**/0.02	150/**0.33**/0.02	4609/**0.33**/0.02	N/A	4609/**0.33**/0.02
Phantom Image Background OD	4276/**1.85**/0.22	3328/**1.85**/1.20	653/**1.89**/0.20	334/**1.85**/0.22	8591/**1.85**/0.21	N/A	8591/**1.85**/0.21
Fibers^d^	4614/**5.1**/0.6	3573/**5.0**/0.6	771/**5.1**/0.6	351/**5.0**/0.6	9135/**5.1**/0.6	892/**5.3**/0.6	8568/**5.0**/0.6
Specks^d^	4614/**3.9**/0.3	3573/**3.9**/0.4	771**/3.9**/0.3	351/**3.9**/0.4	9135/**3.9**/0.3	892/**4.0**/0.3	8568/**3.9**/0.3
Masses^d^	4614/**3.9**/0.5	3573/**3.9**/0.5	771/**4.0**/0.4	351/**3.9**/0.4	9135/**3.9**/0.4	892/**4.3**/0.4	8568/**3.9**/0.4
Total Net Score^e^	4614/**12.4**/1.1	3725/**12.4/**1.1	770/**12.6**/1.1	351/**12.3**/1.1	9309/**12.4**/1.1	891/**13.5**/0.9	8567/**12.3**/1.1
Film Processing: % < 80^f^	0.1 (2/2852)	0.0 (0/2389)	0.0 (0/358)	0.0 (0/262)	0.0 (2/5861)	0.0 (0/166)^h^	0.0 (2/5761)
Darkroom Fog: % > 0.05 OD^g^	4.7 (127/2725)	4.0 (92/2325)	1.3 (4/304)	4.3 (10/233)	4.2 (233/5587)	3.2 (7/220)^h^	4.2 (235/5565)
% Phantom Images with artifacts	69.9 (3226/4614)	69.9 (2498/3573)	69.1 (533/771)	69.8 (245/351)	69.9 (6502/9307)	29.9 (267/892)	73.2 (6270/8568)

a 18 facilities that were inspected between August (during which MQSA inspections of digital equipment began) and December 2005 were also included in the population of digital sites.

b Dose is that reported by the Medical Physicist.

c In mid-2006 FDA ceased equipment radiation measurements for HVL, hence the sample size is smaller than those for the remaining tabulated parameters.

d Object score is reported without artifact subtraction.

e Score includes artifact subtraction.

f Percentage of standard-cycle film processors with a speed less than 80, as determined by the STEP method.

g Percentage of facilities found to have darkroom fog greater than 0.05 net optical density.

h If the facility also operated at least one screen-film mammography unit then the film processing quality and darkroom fog were evaluated.

Although a higher dose translates to higher radiation risk for the patient, MQSA data also demonstrated an increase in benefit as indicated by improved image quality performance. Phantom image background optical density increased substantially from a mean optical density of 1.42 in 1995 to 1.85 in 2006. This finding indicates that film contrast performance should improve because most mammography films currently in use provide optimal image contrast at optical densities approaching 2.0 [14]. Indeed, although there was improvement in all three object groups (Tables 1 and 2), the fibre and mass groups showed the most statistically significant improvement between 1995 and 2006, the visualisations of which are dependent on the contrast performance of the film and therefore sensitive to the background optical density [15]. MQSA requires mammography facilities to maintain a phantom image background optical density of at least 1.2.

Artefacts are a detriment to the clinical value of the mammogram and can arise from many sources including contaminated film cassettes, the film processor, the darkroom environment, improper film handling, and the mammography unit compression paddle, among other sources [16]. Between 1995 and 2006, there was a 10% increase in the incidence of artefacts reported on phantom image films scored by MQSA inspectors from 60% (6,979 of 11,676) of inspected mammography units in 1995 to 70% (6,502 of 9,307) of inspected mammography units. Across all types of facilities, speck-like artefacts were the most frequent type reported, occurring on 58% (6,745 of 11,676) of mammography units inspected in 1995 and on 68% (6,293 of 9,307) of mammography units inspected in 2006. Mass-like artefacts were the least frequently reported artefact type, and occurred on less than 5% of mammography units 1995 (523 of 11,676) and 2006 (323 of 9,307). Improvements in film processing technology, maintenance of mammography equipment, and adherence to acceptable quality control and quality assurance practices can reduce artefacts. However, if film contrast performance improves as indicated by higher background optical densities, then the visualisation of artefacts may also improve. Finally, the training and experience of inspectors can influence the reporting of artefacts.

### Film processing and darkroom fog

Reports have documented substantial improvements in film processing quality by mammography facilities between 1985 and 1997 [2,3]. During this period, extended cycle processing was used by a substantial number of facilities. In 1992, 26% of surveyed facilities claimed to use extended cycle processing, and three quarters of these sites had sub-optimal processing quality for this particular processing cycle [2]. The rate of film processors that facilities claimed to operate in extended cycle mode reached 43% (2,743 of 6,459) in 1995, but by 2006 extended cycle processing nearly vanished. Nearly 97% (5,861 of 6,068) of film processors are presently being operated in a standard cycle mode. Regardless of the processing cycle, MQSA inspection results document that mammography facilities have maintained high quality in film processing. During the first year of inspections 4% (243 of 6,459) of tested film processors (all processor cycles) were found to have a STEP processing speed below the MQSA action limit, and by 2006 this percentage dropped to near zero (3 of 6,068). The high rate of compliance with the MQSA standard for film processing can be attributed to better quality control and quality assurance practices, improvements in film and chemical processing technologies, and a heightened awareness by facilities and the professional community regarding the impact that film processing quality can have on clinical image quality. Whereas film processor control charting merely tracks the drifting of processing quality from a pre-established and possibly arbitrary operating level, the STEP film processor test can provide a benchmark operating point for film processing of optimal quality.

Reducing radiographic film fog in the darkroom to acceptable levels is a simple yet often ignored aspect of quality control. In 1992, 62% of clinical facilities had darkroom fog levels in excess of the current MQSA standard (net optical density no greater than 0.05) [2]. During the first year of MQSA inspections, 11% (778 of 7,030) of inspected darkrooms exceeded this limit, and by 2006, the non-compliance rate dropped to less than 5% (233 of 5,587), with breast clinics showing the highest rate of compliance (Table 2).

### Digital mammography

**Figure 6:**
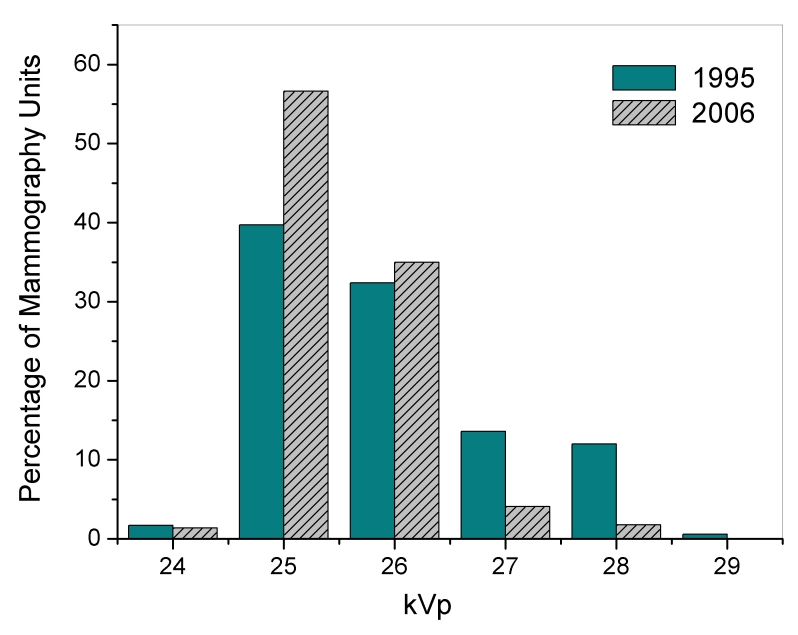
Number of accredited digital mammography (DM) units (circles) and the number of certified digital mammography facilities (squares).

**Figure 7:**
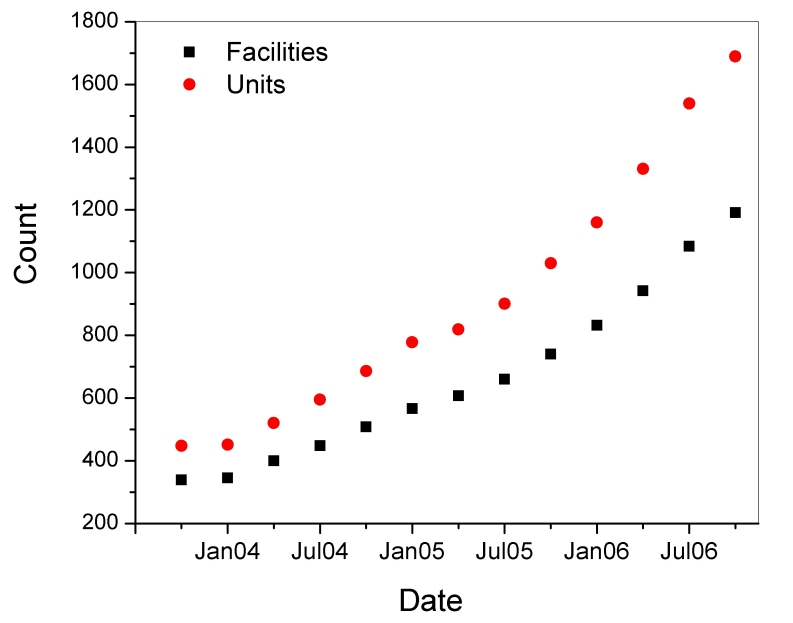
Geographic distribution of screen-film and digital facilities in the U.S. for 2005-2006. A facility was classified as a digital site if it had at least one digital mammography unit. A facility that has digital mammography may also have conventional screen-film technology, and therefore was included in the latter category as well.

Table 2 includes findings of selected parameters for DM and SF units inspected between January and September 2006. DM was first approved in the U.S. in 2000, and currently there are approximately 1,689 DM units in use at 1,191 certified mammography facilities (Figure 6), comprising approximately 14% of the total population of certified mammography facilities. Figure 7 shows a geographical distribution of DM sites and SF sites. Because of the complexities regarding the evaluation of dose and quality assurance/quality control procedures that tended to be specific to each manufacturer, the FDA conducted abbreviated inspections of digital facilities to verify that the manufacturers’ recommended practices were being instituted. MGD was captured from the medical physics survey report, and image quality was evaluated by the FDA inspector using the same standard phantom as that used for screen-film mammography. Data analysis of DM was conducted on inspections that took place between August 2005 and October 2006 in order to include all inspected digital facilities.

### Workload

**Figure 8:**
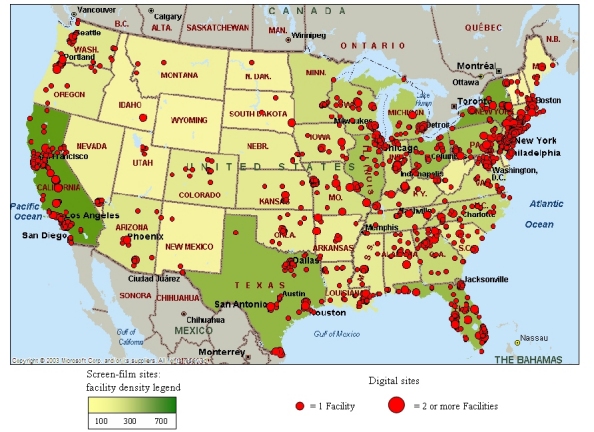
Distributions of facility types for mammography facilities using only screen-film technology (left) and for facilities that had at least one digital mammography unit (right). Data for screen-film is for January to October, 2006. Data for digital mammography is from August 2005 to October 2006.

**Figure 9:**
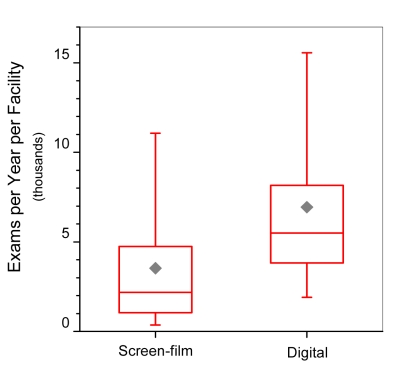
Annual screening exam workload per facility for sites that used only screen-film technology and for sites that had at least one digital mammography unit. Box bottom and top borders are 25^th^ and 75^th^ percentiles respectively, and box contents are 50^th^ percentile (line) and mean value (diamond). Whiskers indicate 5^th^ and 95^th^ percentiles.

The distribution of digital equipment across the different types of facilities is similar to that for screen-film equipment (Figure 8). The majority of digital sites are hospitals; however breast clinics comprise 11% (72 of 633) of digital sites compared with 6% (345 of 6,010) of screen-film sites. Facilities using DM reported an average facility annual workload (6,938 exams per year, N=75), approximately double the average for SF sites (3,524 exams per year, N=1212) (Figures 9).

### Dose and Image Quality

**Figure 10:**
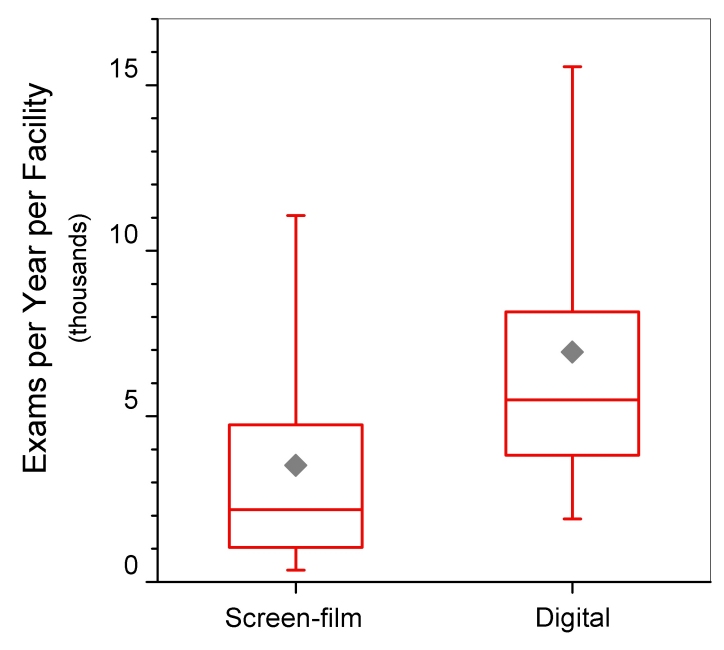
Mean glandular dose for screen-film versus digital mammography units. For both modalities dose was captured from the facility’s medical physics survey report. Box bottom and top borders are 25^th^ and 75^th^ percentiles respectively, and box contents are 50^th^ percentile (line) and mean value (diamond). Whiskers indicate 5^th^ and 95^th^ percentiles.

**Figure 11:**
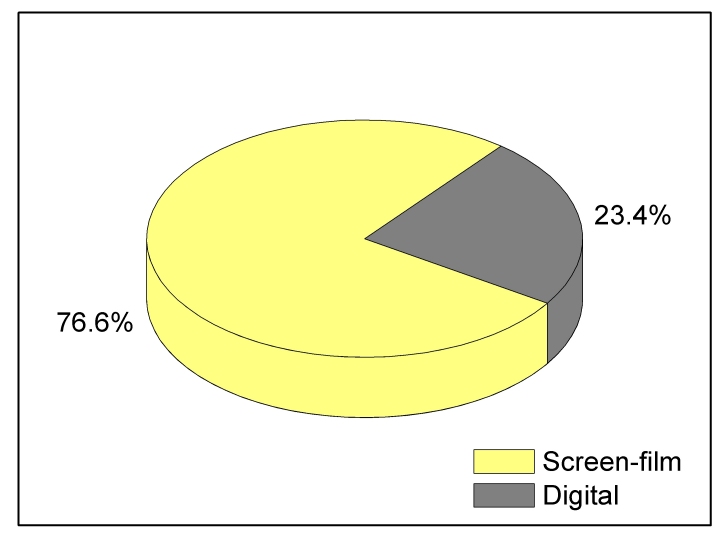
Phantom image-quality score for screen-film versus digital mammography units. Total score without artifact subtraction is reported. Box bottom and top borders are 25^th^ and 75^th^ percentiles respectively, and box contents are 50^th^ percentile (line) and mean value (diamond). Whiskers indicate 5^th^ and 95^th^ percentiles.

Figures 10 and 11 display distributions of MGD and phantom image quality score for facilities using SF and DM. The average MGD for DM units (1.63 mGy) was statistically lower (p < 0.001) than that for SF units (1.80 mGy). Both means are well below the MQSA compliance limit for MGD of 3.0 mGy for a single (craniocaudal) view. The standard deviations for MGD in Table 2 and the dose distributions in Figure 10 highlight the broader range of doses for DM. MQSA inspectors did not collect any dose-related technical factors such as clinically selected kVp, beam quality, or target-filter selection during the inspection of DM units, and therefore it is not possible to compare these parameters between the two technologies.

The MQSA inspector imaged the phantom on the DM unit and scored the image using the same format (hard-copy or soft-copy workstation) as that routinely used by the facility. Sixty-two percent (554 of 889) of DM phantom images were scored on a computer monitor. If the soft-copy image score failed to meet minimum standards, a hard-copy image was evaluated prior to issuing a citation to the facility. Table 2 summarises image quality scores for the three individual object groups (without artefact subtraction), and the total net score, including artefact subtraction. Average image quality score (net score including artefact subtraction) for DM (13.5 objects) was significantly higher (p < 0.001) than for SF (12.3 objects). If the presence of artefacts is not accounted for, the difference between the mean raw total score for SF (12.8 objects) and DM (13.6 objects) decreased (Figure 11), but was still statistically significant (p < 0.001). Most of the contribution to higher total raw scores for DM was from the mass object group (p < 0.001), while the smallest yet also statistically significant (p < 0.001) difference was observed for the speck object group.

Analysis of raw object scores can indicate the overall ability of the system to visualize clinically relevant features, whereas the presence of image artefacts is an indicator of the tendency of the system to superimpose false structures on the clinical image. Seventy-three percent (6,270 of 8,568) of phantom images produced using SF technology were found to have at least one artefact compared to 30 percent (267 of 892) of phantom images produced using DM. For both modalities, the artefact type that was identified most frequently by the inspector was a speck-like artefact, and occurred in 25% (222 of 892) of DM images and 71% (6,102 of 8,568) of SF images. The least frequent artefact type was a mass-like artefact, identified on only 2% (17 of 892) of DM images and 4% (306 of 8,568) of SF images. Specifically for DM, there was a slightly higher occurrence of artefacts for soft-copy review than for the hard-copy format across all object groups, and overall artefacts were observed on 24% (82 of 335) of hard-copy films and on 33% (183 of 554) of soft-copy images. Possible reasons for the much lower incidence of artefacts with DM compared to SF may include the elimination of conventional film processing and related artefacts associated with SF, and any software-based image processing features or other electronic features in DM such as flat-field corrections that may reduce the presence or visualisation of artefacts.

**Figure 12:**
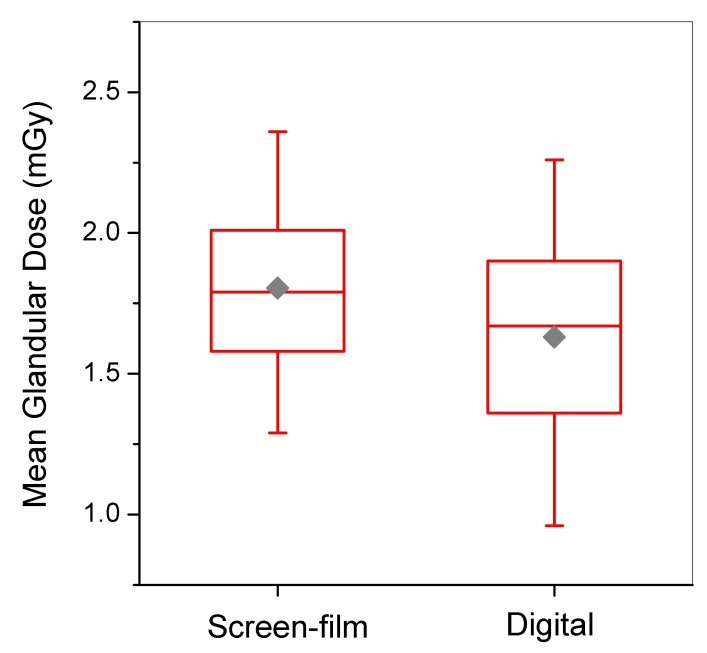
Scatter plots and linear fits of mean phantom image score (without artifact subtraction) versus mean glandular dose. Note that phantom scores are constrained to integer and half-integer values.

Is better image quality associated with higher dose? In screen-film imaging, reducing dose can result in lower image quality scores depending on the sensitometric properties of the film. In DM, the pixel-based signal-noise ratio can be reduced. For both SF and DM, linear regression analysis for a dependence of phantom image quality score (without artefact subtraction) on dose yielded only a weak relationship (correlation coefficient (r) < < 1). However, the null hypothesis- that the slope of the regression line is zero- was rejected (p < 0.001) for both imaging modalities. Testing was also performed for a possible difference in image quality between mammography units that produced doses below 1.0 mGy compared with mammography units having doses greater than 2.0 mGy. These ranges were selected because they exclude the average dose values that have occurred for the populations of inspected facilities between 1995 and 2006. For SF mammography, the average raw total score for doses below 1.0 mGy was 12.3 objects compared with 13.0 objects for doses greater than 2.0 mGy. For DM, the corresponding average image quality scores were 13.1 and 13.8 objects, respectively, for doses below 1.0 mGy and above 2.0 mGy. The difference in average raw total score for the two dose groups is statistically significant (p < 0.001) for both SF and DM. These findings, which characterize aspects of the dose-phantom image quality relationship for a population that is representative of the state of practice, demonstrate that dose has a weak but observable impact on image quality (Figure 12). Haus et al. [15] observed a similar relationship in which phantom image score failure rates were significantly higher for doses below 1.0 mGy compared with doses in the range of 1.5-2.0 mGy.

## DISCUSSION AND CONCLUSIONS

The annual number of screening mammography exams conducted in the U.S. has steadily increased between 1997 and 2006, with hospitals and private practice sites conducting the majority of exams. DM is allowing facilities to increase their exam workloads, and such facilities are estimated to conduct almost a quarter of all mammography exams.

**Figure 13:**
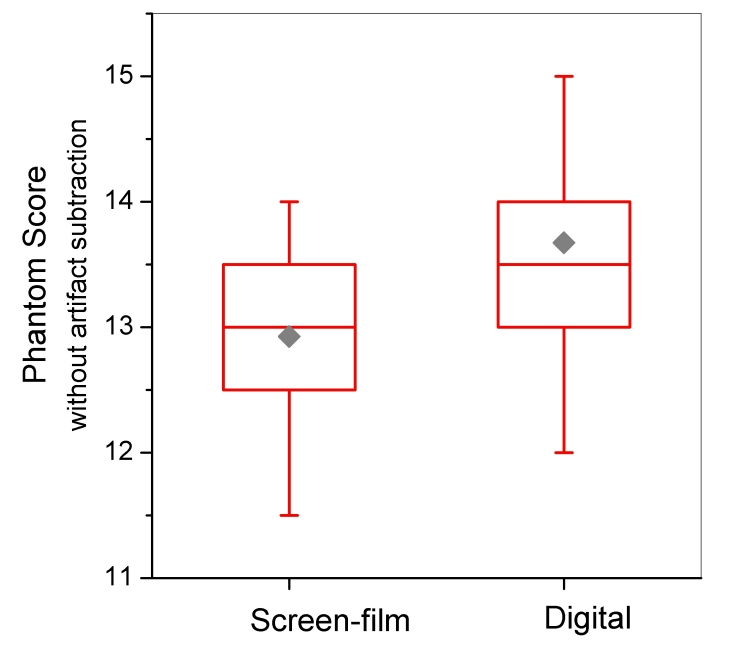
Dose and image quality trends for mammography in the United States. Data were obtained from the following sources. 1974 (dose): Bicehouse HJ. Survey of Mammographic Exposure Levels and Techniques Used in Eastern Pennsylvania. 7th Annual National Conference on Radiation Control, 1975. DHEW Publication (FDA) 76-8026. 1976 (dose): Butler PF, Jensen JE. Breast Exposure: Nationwide Trends; A Mammographic Quality Assurance Program- Results to Date. Radiologic Technology 50(3), 1978; pp 251-257. 1980 (dose): Breast Exposure: Nationwide Trends. In: Internal project progress report. Rockville MD: Bureau of Radiological Health, US Department of Health and Human Services, Food and Drug Administration, 1981. 1985, 1988, 1992 (dose and image quality): Conway BJ, Suleiman OH, Rueter FG, Antonsen RG, Slayton RJ. National Survey of Mammographic Facilities in 1985, 1988, and 1992. Radiology 1994; 191: 323-330. 1995-2006 (dose and image quality): Mammography Quality Standards Act (MQSA) inspection findings. Image Quality scores are reported for following phantoms: 1985: RMI 152 phantom with 'C' insert 1988: RMI 156 phantom with 'C' insert 1992 to present: RMI 156 phantom with 'D' insert (or equivalent)

Screen-film based mammography in the U.S. has matured technically as both imaging equipment performance and facility clinical practices have promoted reduced variability in MGD and indicators of image quality. Figure 13 shows the trends in mammography dose and image quality in the U.S. between the mid-1970s and 2006, and it highlights two aspects of the clinical practice. Prior to the 1990s, changes in the technical aspects of mammography dramatically reduced radiation dose (risk) and improved image quality (clinical benefit). As mammography technology matured and mandatory quality standards were instituted nationally, dose actually increased slightly as the professional community optimised image quality to a stable level.

Average MGD in the U.S. currently is higher than those reported in surveys conducted in several other countries. Young et al. reported for the UK in 2001 and 2002 an average MGD of 1.42 mGy for a slightly larger compressed breast thickness of 45 mm in their model [17]. They also reported a similar increasing trend in dose in the breast model compared to a previous survey conducted there in 1997 to 1998. A survey conducted in the Netherlands also reported lower doses; however breast doses were based on tissue glandularity and an average compression thickness between 5.4 cm (average MGD of 1.04 mGy) and 6.2 cm (average MGD of 1.63 mGy) depending on locality [18]. Jamal et al. reported an average MGD from a survey in Malaysia conducted between 1999 and 2001 of 1.23 mGy using the RMI model 156 phantom [19] and reported an average phantom image background optical density (1.28) well below the value reported in this paper for the U.S. in 2006. Image quality indicators should be considered before concluding that there is a clinical benefit to administering lower doses.

Film processing quality in the U.S. has continued to improve and standard cycle processing has become the de facto standard in mammography. In 1995, over 25% (957 of 3,717) of tested (standard cycle) film processors were operating at a speed below 90 compared with only 1.3% (73 of 5,861) of film processors tested in 2006. This observation and the fact that dose has actually increased on average both indicate that facilities have increasingly directed their efforts toward improving clinical benefit.

Although screen-film based mammography is still the dominant imaging format, data reported in this paper suggest that DM can offer at least comparable and possibly superior image quality performance with lower mammographic dose. This study did not evaluate additional features provided by digital-based imaging such as computer-assisted manipulation of the image. Although these findings suggest that DM is currently producing better image quality as indicated by higher scores for test objects including significantly fewer artefacts, it remains to be shown that they are clinically significant. The results of the ACRIN DMIST trial [20] indicating that DM is not superior to SF imaging for all patients is consistent with the conclusion that DM at this time is still a maturing technology not yet definitively superior to screen-film mammography.
